# Asymptomatic microperforated transverse vaginal septum presenting with primary infertility: a rare form of mullerian anomaly

**DOI:** 10.4274/tjod.galenos.2019.32956

**Published:** 2019-07-03

**Authors:** Erbil Doğan, Onur Yavuz, Canan Altay, Samican Özmen

**Affiliations:** 1 Dokuz Eylül University Faculty of Medicine, Department of Obstetrics and Gynecology, İzmir, Turkey; 2 Dokuz Eylül University Faculty of Medicine, Department of Radiology, İzmir, Turkey

**Keywords:** Transverse vaginal septum, infertility, congenital malformation

## Abstract

Transverse vaginal septum is a rare type of mullerian anomaly resulting from failure of the canalization of the vaginal plate at the point where the urogenital sinus meets the mullerian duct and usually presents at menarche with symptoms of outflow tract obstruction. Instead, patients with a perforated septum often have normal menses and usually present with difficulties with intercourse or infertility. A 24-year-old patient with 5 years of infertility is reported. Following assessment, isolated microperforated transverse vaginal septum (U0C0V3 according to the new classification system of the European Society of Human Reproduction and Embryology/European Society for Gynaecological Endoscopy for congenital uterine anomalies) was detected with no additional urogenital anomaly and simple excision of the septum and end-to-end vaginal anastomosis was performed. The patient became pregnant spontaneously 2 months after the operation when sexual intercourse was permitted. Transverse vaginal septum, which presented itself with no clinical findings and only primary infertility, is discussed with a review of the existing literature.

## Introduction

Transverse vaginal septum is a rare type of mullerian anomaly resulting from failure of the canalization of the vaginal plate at the point where the urogenital sinus meets the mullerian duct. Its incidence is estimated as 1/70,000 females, making it one of the rarest anomalies of the female genital tract^(1)^. Imperforate septum usually present early in adolescence with obstructed menstruation. However, patients with perforate septum often have normal menses and usually present with difficulties with intercourse or infertility. The exact localization and the thickness of the septa vary, but are more frequent in the upper vagina and mostly less than 1 cm in thickness^(2)^. In this article, we present a patient with a perforated transverse septum who presented with primary infertility and underwent treatment of the septum.

## Case Report

**Figure 1:**
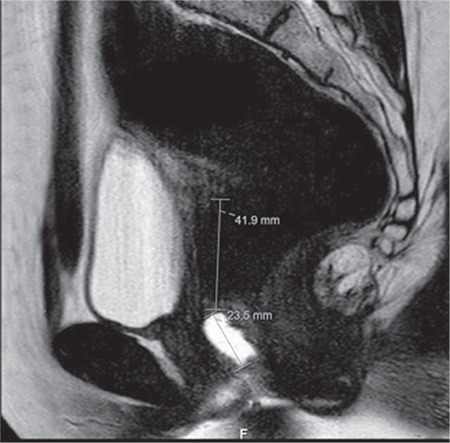
Sagittal view of the magnetic resonance imaging scan after gel injection into the vagina showing the exact level of the septum (A: distance from introitus to septum: 23.5 mm; B: distance from septum to posterior vaginal fornix: 41.9 mm)

A 24-year-old patient with 5 years’ infertility was admitted to outpatient clinic for treatment. She had no systematic disease. Her menarche started when she was aged 12 years and her menstrual cycle was regular. The patient did not describe any symptoms such as dysmenorrhea, pelvic pain or dyspareunia, nor did she have problems during sexual intercourse. Secondary sex characters were found to be compatible with her age. There was no pathology in the external genitalia. On speculum and digital examinations, a blind-ending vagina was detected. There was no orifice and the cervix was not visible. Transvaginal ultrasound revealed normal uterine and adnexal anatomy. Hematocolpos and hematometra were not detected. Therefore, a microperforated transverse vaginal septum was suspected. There were no other associated urogenital congenital anomalies of the upper and lower abdomen on ultrasound. In order to confirm the diagnosis, abdominopelvic magnetic resonance imaging (MRI) was performed following ultrasound gel injection into the proximal vagina, which showed a transverse vaginal septum at the middle part of the vagina, 23-mm distant from the introitus. The distance between the anterior fornix in the superior and this level of septum was 41 mm (Figure 1).

During surgery, transverse vaginal septum was excised circularly, and the proximal and distal parts were anastomosed. A normal-appearing cervical ostium was then visible. The vaginal tissue around the fibrotic tissues was sutured in a purse string fashion with late absorbable sutures. Diagnostic hysteroscopy and laparoscopy were also performed for the investigation of infertility. On hysteroscopy, a normal endometrial cavity with two normal tubal ostial orifices and a normal endocervical canal were seen. Laparoscopy also revealed normal pelvic anatomy and a methylene blue dye test showed bilateral patent tubes.

After the operation, to prevent vaginal stricture the patient wore a vaginal mold prepared with a sterile condom filled with tampons. This mold was changed every two days and repeated 5 times totally. Prophylactic antibiotics were given to prevent vaginal infection. Upon follow up, no vaginal infections, strictures or any gynecologic pathologies developed in the postoperative period. Wound healing was complete and sexual intercourse was permitted 8 weeks after the operation. She became pregnant spontaneously the next month and had 21 weeks of normal pregnancy at the time of writing this article.

## Discussion

One of the rare causes of primary infertility is transverse vaginal septum. It results from either incomplete canalization of the vaginal plate or failure of the paramesonephric ducts to meet the urogenital sinus^(2)^. There are few data available in the literature about the classification or the surgical management of transverse vaginal septae. Additionally, the short or long-term results following surgical treatment are also not very clear^(3)^.

The classification is made using the new European Society of Human Reproduction and Embryology/European Society for Gynaecological Endoscopy consensus in 2013 and transverse vaginal septum was accepted as the V3 subgroup among the mullerian duct anomalies^(4)^. Our case was classified as U0C0V3 because our patient had no associated uterine or cervical anomalies.

Perforated-type transverse vaginal septa is usually asymptomatic until adolescence or adulthood because these patients have no outflow obstruction. As in our case, they may present with infertility or sometimes with coital problems^(5)^. They may also be diagnosed incidentally during vaginal examination. The reason for infertility is not exactly clear, but microperforated septum may be an obstacle for the passage of sperm. Clinical examination, ultrasound, and MRI may all be used in the diagnosis and preoperative planning. Treatment involves surgical resection of the septum and anastomosis of the proximal and distal parts. The main goal during surgery must be to maintain the continuity of the vaginal epithelium and restoration of normal vaginal caliber and length.

Transverse septums can be seen at different locations of the vagina. According to the study of Williams et al.,^ (6)^ 15-72% of the transverse vaginal septum cases are seen at the distal end of the vagina, 22-40% are seen at the mid-vagina, and 6-45% are seen at the proximal part. Our patient had a mid-vaginal septum. Diagnosis and the treatment plan should be made after performing a clinical examination, ultrasonography, and MRI. The treatment can be made with vaginal, laparoscopic, and abdominoperineal approaches according to the localization and the thickness of the septum. Complication rates are low if the septum is located at the distal part of the vagina and it is a thin, perforated septum. If the septum is at the mid-upper part of the vagina and it is not thicker than 2 cm, laparoscopy would be the correct treatment approach. If the septum is thicker than 2 cm, the abdominoperineal approach is necessary; however, complication rates are high^(7)^. These complications are mostly vaginal stenosis and reobstruction. In the study of Joki-Erkkilä and Heinonen^(7)^ two of three patients with isolated transverse septae had reobstruction and needed reoperation despite their septal thicknesses being less than 1 cm. Therefore, to prevent such obstruction, regular postoperative dilator therapy is essential. Additionally, early coitus after complete healing must also be advised. We also performed diagnostic laparoscopy and hysteroscopy after excision of the septa, which showed an anatomically normal uterus, ovaries, and patent tubes, and also a normal endometrial cavity. Laparoscopy could have been skipped because we performed an MRI scan before the operation and it showed normal findings; however, we performed a laparoscopy to document tubal patency and to rule out endometriosis.

## Conclusion

Transverse vaginal septum is a rare mullerian tract anomaly and selected patients can be treated by simple excision and anastomosing the proximal and distal vaginal tissue. It is safe, effective, and easy to perform. The recurrence and complication rates vary due to the location and the thickness of the septum. Surgical resection of the septum results in successful restoration of the genital tract anatomy and allows normal fertility.
